# Memory T cells: promising biomarkers for evaluating protection and vaccine efficacy against leishmaniasis

**DOI:** 10.3389/fimmu.2024.1304696

**Published:** 2024-02-26

**Authors:** Mahmoud Nateghi-Rostami, Yahya Sohrabi

**Affiliations:** ^1^ Department of Parasitology, Pasteur Institute of Iran, Tehran, Iran; ^2^ Department of Cardiology I-Coronary and Peripheral Vascular Disease, Heart Failure, University Hospital Münster, Westfälische Wilhelms-Universität, Münster, Germany; ^3^ Department of Medical Genetics, Third Faculty of Medicine, Charles University, Prague, Czechia

**Keywords:** leishmaniasis, memory CD4^+^ T cells, vaccine, tissue resident memory, biomarkers

## Abstract

Understanding the immune response to *Leishmania* infection and identifying biomarkers that correlate with protection are crucial for developing effective vaccines. One intriguing aspect of *Leishmania* infection is the persistence of parasites, even after apparent lesion healing. Various host cells, including dendritic cells, fibroblasts, and Langerhans cells, may serve as safe sites for latent infection. Memory T cells, especially tissue-resident memory T cells (T_RM_), play a crucial role in concomitant immunity against cutaneous *Leishmania* infections. These T_RM_ cells are long-lasting and can protect against reinfection in the absence of persistent parasites. CD4^+^ T_RM_ cells, in particular, have been implicated in protection against *Leishmania* infections. These cells are characterized by their ability to reside in the skin and rapidly respond to secondary infections by producing cytokines such as IFN-γ, which activates macrophages to kill parasites. The induction of CD4^+^ T_RM_ cells has shown promise in experimental immunization, leading to protection against *Leishmania* challenge infections. Identifying biomarkers of protection is a critical step in vaccine development and CD4^+^ T_RM_ cells hold potential as biomarkers, as their presence and functions may correlate with protection. While recent studies have shown that *Leishmania*-specific memory CD4^+^ T-cell subsets are present in individuals with a history of cutaneous leishmaniasis, further studies are needed to characterize CD4^+^ T_RM_ cell populations. Overall, this review highlights the importance of memory T cells, particularly skin-resident CD4^+^ T_RM_ cells, as promising targets for developing effective vaccines against leishmaniasis and as biomarkers of immune protection to assess the efficacy of candidate vaccines against human leishmaniasis.

## 1 Introduction

Leishmaniasis, a disease caused by the protozoan parasites of the genus *Leishmania*, is a major worldwide health concern. The endemicity of the disease has been established in approximately 100 countries across the globe, with diverse clinical forms, including cutaneous (CL), mucocutaneous (MCL), visceral (VL), and post-kala-azar dermal (PKDL) leishmaniasis (1). CL typically manifests as a naturally healing skin lesion, yet there are exceptional cases where it does not follow the expected healing timeline and remains unresponsive to repeated antimonial treatments. Despite immense efforts to manage the disease through various approaches such as chemotherapy and vector/reservoir control, success rates remain limited in most endemic regions. It is important to note that, as of now, there is no vaccine accessible for any form of human leishmaniasis (2, 3).

While a wealth of information has been amassed regarding the factors influencing the pathogenesis of *Leishmania* infection and the immune responses mounted by the host, encompassing both animal models and human patients, there remains a notable absence of well-defined immune biomarkers that can reliably indicate recovery and protection in human leishmaniasis (4). It is well known that in a mouse model of leishmaniasis, the specific immune response elicited following infection with *Leishmania* parasite significantly influences the ultimate disease outcome, whether it leads to recovery and protection or exacerbation of the disease (5). Experimentally, resolution of the leishmanial lesion due to *L. major* infection in resistant mice is followed by the development of a lifelong immunity to reinfection, shown by cellular immune response and delayed-type hypersensitivity (DTH). It is widely acknowledged that individuals who develop CL through natural infection due to sand fly bites or through leishmanization experience robust protection against the development of additional lesions. This underscores the rationale for the development of a leishmaniasis vaccine (6, 7).

A safe vaccine with high efficacy that provides protection against both Old World and New World *Leishmania* species causing CL or VL would undoubtedly benefit the people in certain economically deprived areas of the world. During the last few years, a myriad of potential *Leishmania* vaccine candidates have been put forth, spanning various types such as live organisms, attenuated strains, genetically engineered parasites, inactivated forms, and subunit or fusion proteins. However, most of them have been evaluated in preclinical phases and shown immunogenicity in animal models [reviewed in (8, 9)] and only a limited number of candidates have a clear and timely path toward evaluation in clinical trials (Nateghi Rostami, et al., under review) (10). ChAd63-KH is a replication defective simian adenoviral vaccine expressing a synthetic gene (KH) and encoding two *Leishmania* proteins kinetoplastid membrane protein 11 (KMP-11) and hydrophilic acylated surface protein B (HASPB). It has progressed through a phase I human trial in healthy UK volunteers and a single intramuscular vaccination in Sudanese patients with persistent PKDL in phase IIa and phase IIb trials to evaluate therapeutic efficacy (11, 12). There are few other vaccine candidates that are in late-stage preclinical development. *Lm*Cen^−/−^ cultures have been produced under GMP conditions, and phase I trials are being planned in the USA and India. The use of mRNA vaccine platforms for leishmaniasis has not been explored yet (13), but mRNA vaccines encoding *Leishmania* recombinant LEISH-F2 and LEISH-F3 antigens are in the late preclinical evaluation stage. This mRNA vaccine builds upon the promising outcomes observed in human efficacy trials of the LEISH-F1, F2, and F3 vaccines. LEISH-F vaccines consist of fusion recombinant proteins with adjuvants and were among the first second-generation vaccines to undergo human testing (14).

In addition to humans, various animal species including rodents and canids can serve as reservoirs or infected with *Leishmania* spp (15). as seen in the zoonotic cycle of CL due to *L. major* and of VL due to *L. infantum.* High infection rates in dogs, which usually live close to humans, facilitate the domestic transmission of *L. infantum* to humans, as indicated by several studies reporting a high incidence of canine VL in endemic regions of human VL (16, 17). Regarding canine VL, four vaccines have been licensed since 2000s, two in Brazil (Leishmune^®^ and Leish-Tec^®^) and two in Europe (CaniLeish^®^ and LetiFend^®^). The production and marketing license of Leishmune^®^ vaccine was withdrawn due to lack of effectiveness. Some concerns regarding effectiveness and potential infectiousness remain for all of these vaccines (18).

Despite advances in experimental and preclinical vaccine research, one reason for the lack of an approved human leishmaniasis vaccine might stem from our incomplete comprehension of the factors that signify protective immunity (4). Additionally, animal models for leishmaniasis often do not precisely reproduce the human form of the disease, and the translation of findings from experimental animal models to humans is not always confident (Nateghi Rostami, et al., under review) (19).

## 2 T lymphocyte subsets in leishmaniasis

The effector T cells (T_EFF_) refer to a collection of cells comprising various T-cell types, including both CD4^+^ and CD8^+^ T cells, which actively respond to antigenic stimulations. Following the differentiation of an effector T cell in the lymphoid tissue, an effector T cell locates target cells presenting the MHC:peptide complex. CD8^+^ T cells recognize peptide fragments from cytosolic pathogens, bound to MHC class I molecules at the cell surface (20). Cytotoxic CD8^+^ T cells can limit intracellular infections by direct cytolysis via perforin/granzymes and Fas/FasL interactions or by activating immune responses through production of cytokines like IFN-γ. On the other hand, both CD4^+^ Th1 and CD4^+^ Th2 cells recognize antigen fragments from intracellular vesicles displayed on the cell surface by MHC class II molecules. In murine experimental *L. major* infection, CD4^+^ T cells differentiate into Th1 or Th2 subpopulations that determine infection outcomes. Resistant C57BL/6 inbred mice develop a strong Th1 response with high interferon (IFN)-γ production that promote healing of *L. major* lesions and parasite clearance, while susceptible BALB/c inbred mice develop a Th2 response with high IL-4 production that cause non-healing lesion and systemic dissemination of *L. major* infection and eventual death of the animal (21).

Besides the host’s genetic background, outcomes of the disease are influenced by the parasite strain, infection route, and parasite dose (22). For example, in contrast to *L. major* infection, *L. braziliensis* infection in BALB/c mice triggers an effective immune response resulting in lesion healing that coincides with parasite persistence in draining lymph nodes. Subcutaneous inoculation of a low dose of *L. infantum* induces a Th1 response accompanied by a low parasite burden, while high-dose infection results in a Th2 or mixed Th1/Th2 response and a high parasite burden in spleen and lymph nodes. Although Th1/Th2 polarized response is well established in the C57BL/6 and BALB/c mice strains, this paradigm sometimes falls short in explaining host immunity, especially in humans (23, 24) (Nateghi-Rostami, et al, under review).

Another T-cell population, Th17 cells, expressing skin and mucosal homing receptors such as CCR4 and CCR6, is involved in neutrophil and monocyte recruitment, producing cytokines (IL-17, IL-22, and IL-23) and providing antimicrobial immunity at epithelial and mucosal surfaces (25). The contributive role of Th17 cytokines in either protection against infection or exacerbation of the disease has been suggested in animal models of *L. major* and *L. donovani* infections (26–28). It is claimed that IL-17 and IL-22 may synergize with Th1 cytokines for protection against human VL due to *L. donovani* (29); in our study, patients with active VL due to *L. infantum* exhibit higher IL-22 production, decreasing after VL healing. No significant difference was found in IL-17 production *in vitro* between active and healed CL or VL patients (30, 31). It was suggested that Th17 cell population is suppressed by regulatory T (T_REG_) cells through TGF-β and IL-35 production during chronic VL (32).

CD4^+^CD25^hi^FOXP3^+^ T_REG_ cells are known for their lymphocyte-suppressing mechanisms, including regulatory cytokine expression, such as IL-10, during inflammatory and infectious diseases. In both naive mice and humans, T_REG_ cells make up as much as approximately 5%–10% of the peripheral CD4^+^ T-cell population. Their primary function is to suppress various potentially harmful immune responses *in vivo*, principally reactions targeting self-antigens.

In **Table 1**, a summary of human and mouse studies on the contribution of different T-cell subsets in the immunity against leishmaniasis is provided.

**Table 1:** Cells involved in the immunity against leishmaniasis.

T cell type	Mice studies	Ref.	Human studies		Ref.
			Old World	New World	
**CD4^+^ Th1/Th2 cells**	Th1 response is associated with resistance and control of infection. Th2 response is associated with progressive non-healing disease.	(21) (5)	Th1/Th2 dichotomy is defined but the association with clinical outcome is not established. A mixed Th1/Th2 in non-healing CL cases (*L. tropica*) and a Th2 response in treatment non-responder CL patients (*L. tropica*) A mixed Th1/Th2 in human VL.	A mixed Th1/Th2 response at early lesion development (*L. braziliensis*) Increase in the frequency of CD4^+^ Th1 and CD8^+^ T cells (*L. panamensis*)	(33) (34) (35) (36) (37) (38) (39)
**CD8^+^ T cells**	Contribution in adaptive immunity in cooperation with CD4^+^ T cells. CD8^+^ T cells depletion in BALB/c mice cause IFN-γ reduction and increase in parasite burden. IFN-γ producing CD8^+^ T cells required to revert Th2 response in C57BL/6 mice challenged with low dose of *L. major.*	(40) (41) (42)	Role in IFN-γ production in subjects with history of ZCL	High frequency of IFN-γ producing CD8^+^ T cells in lesions and in PBMC culture of active CL cases. Probably exacerbating role in lesion development.	(43) (44) (34) (45)
**T_REG_ cells**	Natural CD25^+^FoxP3^+^ T_REG_ cells responsible for suppressing immune response in infection site. CD25^-^FoxP3^-^IL-10 producing T_REG_ cells prevent sterile cure and delay healing. CCR5-dependent homing of naturally occurring CD4^+^CD25^+^ T_REG_ cells. The role of IL-10 in the persistence of *L. major* in the skin after healing. Both CD4^+^CD25^+^FoxP3^+^ and CD25^-^Foxp3^-^ (Tr1) T_REG_ cells as the source of IL-10 production in *L. infantum* infection.	(46) (47) (48) (49) (50) (51) (52)	The frequency and function of CD25^+^FoxP3^+^ T_REG_ cells in healed CL cases is comparable to asymptomatic subjects. Elevated levels of IL-10 in human VL. Both CD4^+^CD25^-^Foxp3^−^ T cells and CD4^+^CD25^+^FoxP3^+^ as the source of IL-10 and TGFβ production in *L. donovani*-infected patients, contributing in the pathogenesis of VL.	The absence of IL-10 with increased levels of proinflammatory cytokines cause exacerbating lesion development. Accumulation of T_REG_ cells around dermal lesions is shown and impaired function of T_REG_ cells in inhibition of IFN-γ secretion contributes in pathogenesis. Reduced percentage of T_REG_ cells in blood but higher in lesion.	(53) (54) (55) (56) (57) (58) (59) (60) (61)
**Th17 cells**	High levels of IL-17 in BALB/c mice infected with *L. major*. IL-17 deficiency associated with better control of disease.	(27) (28)	No significant difference between active vs. healed CL cases in the production of IL-17.	Th17 cells exist around mucosal lesions. IL-17 levels correlate with inflammatory response in CL and MCL lesions.	(62) (63) (30)
**T_RM_ cells**	CD4^+^ T_RM_ cells are identified in immune mice, produce IFN-γ, and are parasite independent. *Lm*Cen^−/−^ cause less parasite burden, CD4^+^ T_RM_ substantial production of IFN-γ and Granzyme B in skin. CCL2 and CCL7 by the activated T_RM_ cells. IFN-γ secreted by the skin T_RM_ induces iNOS production by monocytes and enhances protection CD4^+^CD69^+^ T_RM_ cells located in cutaneous *Leishmania* lesions. Development of immune memory subsets in the liver-resident T-cell population in response to *L. infantum* antigens.	(64, 65) (66) (67) (68)	——	——	
**T_EM_/T_CM_ cells**	CD4^+^ T_EM_ and parasite-independent T_CM_ cells are characterized. Immunization of mice with *Ld*Cen^−/−^ induced CD4^+^CD62Lhi, CCR7+ T_CM_ cells, and CD8^+^CD62L^+^CD27^+^ T_CM_ cells	(69) (70) (71) (72)	IFN-γ producing T_EM_ and IL-2 producing T_CM_ are identified in HCL subjects. CD8^+^ memory populations are identified, T_EM_ as the most frequent in HCL subjects.	High frequency of CD4^+^ T_EM_ and CD8^+^ T_EMRA_ cells in CL cases. High frequency of CD4^+^ and CD8^+^ T_EM_ cells in the skin lesions and blood of CL patients. Highlighted role for CD8^+^ T_EM_/T_CM_ cells after treatment in CL patients. Lower frequency of blood CD4^+^CD45RO^+^ memory cells in symptomatic VL patients. Increase in CD4^+^ and CD8^+^ T_CM_ cells, producing IL-2, TNF-α, and IFN-γ, in blood of VL patients following treatment.	(73) (74) (44) (34) (75) (76) (77) (78)

## 3 Persistent parasites and concomitant immunity

### 3.1 The host cells

*Leishmania* parasites survive within the host cells, primarily macrophages, and employ a range of immune evasion strategies to establish and maintain infection (79). The parasites transform into amastigotes within macrophages and replicate continuously, eventually leading to host cell death and rupture. Microbicidal mechanisms of macrophages, particularly reactive oxygen species (ROS) and nitric oxide (NO) productions, play a crucial role in eliminating intracellular parasites (80–82). Different signals such as IFN-γ, CCL2 [chemokine (CC-motif) ligand 2; also known as monocyte chemoattractant protein-1 or MCP-1], and MIP-1α (macrophage inflammatory protein-1 alpha or CCL3) mediate macrophage activation for NO release and subsequent parasite clearance (83). In the evasion mechanisms, some cell surface molecules of *Leishmania*, such as gp63, are involved (84, 85). *Leishmania* promastigotes are exposed to the complement system proteins in the blood and C3b is fixed to the surface of metacyclic parasites via LPG (lipophosphoglycan), but assembly of the C5b-9 attack complex is inhibited, as a result of phosphorylation activity that involves both classical and alternative pathways of the complement system. *Leishmania* promastigotes possess a cell surface protein kinase activity, and as they undergo transformation into metacyclic forms, there is an increase in both the activity of protein kinases and the phosphorylation of protein substrates in the parasite (86). Furthermore, *Leishmania* amastigotes inhibit several host cell phosphorylation signaling pathways (87, 88) and avoid superoxides during phagocytosis by interfering with NADPH oxidase assembly (89). For example, macrophages infected with *L. donovani* displayed impaired capacity to initiate phosphorylation in downstream molecules of Janus kinase (JAK)-signal transducer and activator of transcription (STAT) signaling pathways including STAT-1, JAK1, and JAK2 in response to IFN-γ (90). Downregulation of Toll-like receptors (TLRs) and JAK-STAT signaling pathway genes has also been observed in NK cells obtained from diffused CL (DCL) patients caused by *L. mexicana*, further highlighting the adaptability of *Leishmania* parasites (91). Recently, it was shown that *Leishmania* parasites use kinetoplast DNA to grow inside infected macrophages by taking advantage of the macrophage’s signaling pathway involving Cyclic GMP-AMP synthase (cGAS), stimulator of interferon genes (STING), and TANK-binding kinase 1 (TBK1) for their own benefit (92).

### 3.2 Parasite persistence in CL

Parasite persistence has been documented in both human and mouse models of *Leishmania* infection, but the specific host cells responsible for this prolonged persistence remain uncertain (93, 94). Various host cells, including dendritic cells (DCs), fibroblasts, and Langerhans cells (LCs), may serve as safe sites for latent infection (95–97). In mice resolved from *L. major* infection, viable parasites were found in both macrophages and DCs obtained from the draining lymph nodes (95), but only DCs have the ability to present endogenous parasite antigen to T cells. *In vivo* tracking analysis suggested that the infected DCs found in the lymph nodes were traced back to LCs that had previously migrated from the skin (95) and *L. major*-containing LCs were detected in infected mice skin (96). On the other hand, it was shown that fibroblasts play a substantial role as the host cell harboring a significant number of persisting parasites within the draining lymph nodes of mice following the resolution of cutaneous lesions caused by *L. major* infection (97). Interestingly, the infected fibroblasts failed to eradicate *Leishmania*, unlike infected macrophages. This suggests that fibroblasts might potentially serve as a secure host for the parasites during chronic infections. *Leishmania* amastigotes, residing within parasitophorous vacuoles (PVs) in host cells, rely on essential nutritional elements, including amino acids, purines, and lipids, to support their sustained growth and survival (98).

Belkaid’s group demonstrated that the persistence of *L. major* in the skin of resistant C57BL/6 mice, even after apparent healing, is regulated by an inherent population of CD4^+^CD25^+^ T_REG_ cells (49). In *L. major* infection, CD4^+^CD25^+^ T_REG_ cells recruit in dermis and, with or without IL-10 contribution, inhibit effector T cells from eliminating the parasite at the infection site (48). It was shown that following establishment of *L. major* infection in dermal regions, CCR5 plays a pivotal role in homing CD4^+^CD25^+^ T_REG_ cells to the infected sites, thereby facilitating the parasite’s successful homing and enduring presence in the host (50). Even if the parasite is effectively eliminated in secondary infection sites, occurrence of *Leishmania* superinfection may reactivate the primary infection. This reactivation is orchestrated by T_REG_ cells, which hinder the activation of effector memory responses (99). Both CD4^+^CD25^+^FoxP3^+^ and Foxp3^−^ T_REG_ cells were identified at the lesion site as the source of IL-10 production in the mouse model of *Leishmania* infection (99, 100).

Similarly in human leishmaniasis, T_REG_ cells were characterized in skin lesions of patients with CL, PKDL, and MCL caused by different New World and Old World *Leishmania* species, suggesting a regulatory role of these cells through IL-10 production that contributes to the parasite persistence (53, 76, 101, 102).

### 3.3 Parasites persistence in VL

In the VL infection model, parasite persistence has been shown and the mechanisms of host immunity and immune response evasive mechanisms of the parasite have been discussed (103, 104). *L. infantum* parasites have been shown to persist in dogs in ulcerative skin lesions at primary sand fly bite sites and also at distal sites even after 6 months (105), and under experimental conditions, sand flies could efficiently obtain parasites upon feeding on lesions (105).

In a model of VL infection, it has been suggested that transition of the inflammatory milieu to a regulatory milieu, shown by the increased B-cell activity and IL-10 levels, is associated with the establishment of chronic infection and accumulation of *Leishmania* parasites in the skin (104). In human leishmaniasis, IL-10 plays a central role in the course of the immune response in VL, contributing to the chronicity of the disease through suppression of host immunity and effector T-cell functions (59, 106, 107). Indeed, it was shown that elevated levels of plasma IL-10 and IL-10 expression by keratinocytes indicate the likelihood of subsequent PKDL development in VL patients following treatment (108).

Both CD4^+^CD25^+^Foxp3^+^ and CD25^−^Foxp3^−^ (Tr1) T_REG_ cell populations were found as the source of IL-10 production in *L. infantum* infection in the mouse model (51, 52). Similarly, in dogs as the reservoirs of zoonotic VL, while both CD4^+^ and CD8^+^ T cells expressing CD25^+^FoxP3^+^ were found in the blood and bone marrow of infected animals, antileishmanial therapy caused an increase of CD4^+^CD25^+^FoxP3^+^ T cells in all tissues, ensuring the parasite’s survival and completion of the *Leishmania* life cycle (109).

Similar to mouse findings, controversial studies in *L. donovani*-infected patients introduced both CD4^+^CD25^−^Foxp3^−^ T cells and CD4^+^CD25^+^FoxP3^+^ as the major source of IL-10 and TGFβ productions, contributing in the pathogenesis of human VL (59–61).

### 3.4 Concomitant immunity

Concomitant immunity, described as the immunological memory that is developed when a host is reinfected while presently hosting an initial infection caused by the same pathogen, plays a vital role in controlling *Leishmania* infections. Usually, the permanent survival of pathogens within a host result in the development of potent immunity against subsequent infections. In C57BL/6 mice, following resolution of a primary *Leishmania* infection, mice develop immunity against subsequent challenge infections while concurrently carrying a low level of persistent parasites, which continue to replicate within the host cell. The balance between parasite destruction and survival results in a relatively constant, low parasite count (110). It has been suggested that *Leishmania* parasite replication occurs continually in persistent infections, with most parasites found within activated APCs. Parasites inside iNOS^+^ APCs displayed typical morphology and intact genome compared to parasites within iNOS^−^ cells. This implies that these parasites might possess an unexpected resistance to NO. Based on this study, a model of persistent parasite is proposed in which a population of rapidly replicating *Leishmania* acts as a permanent reservoir, whereas another population with slow or no replication rate is destroyed in APCs, leading to induction of immune response (110). Experimental investigations have revealed that following the resolution of leishmanial lesions, short-lived T-bet^+^ and Ly6C^+^ circulating T_EFF_ offer protection against future challenges but cannot be sustained in the absence of persistent parasites (111). In a chronic *L. major* infection model in resistant C57BL/6 mice, it was shown that CD44^+^CD62L^−^T-bet^+^Ly6C^+^ effector T cells upon adoptive transfer were reactivated by secondary challenge, homed to the skin, and mediated concomitant immunity, shown by high IFN-γ production and inducing protection (111). It seems that the presence of persistent parasites is crucial for maintaining immunity against *Leishmania*, considering that T_EFF_ cells need continuous stimulation to provide protection against reinfection. Supporting this hypothesis are the studies indicating that complete parasite elimination results in the loss of protection in infected mice (49, 112), probably due to depletion of the reservoir of circulating T_EFF_ cells (113). Of note, in the absence of persistent parasite, it remains unclear whether a minor population of memory T cells persists, but their numbers are insufficient for providing protection, or if memory T cells indeed develop but are restricted due to the absence of IL-10 production (114, 115). In concomitant immunity, the time of effector Th1 response at the secondary challenge site on the skin is one of the most significant factors in eliminating parasites, which is linked to a change in how *Leishmania* influences the behavior of monocytic host cells. In experimental *L. major* infection, CXCR3-dependent recruitment of Ly6C^+^ Th1 effector cells and early interactions with phagocytes were shown to be essential for concomitant immunity and preventing the establishment of the pathogen (116).

Increasing the number of effector T cells to control *Leishmania* infection during the acute phase of VL typically results in a subsequent severe loss of effector cells accompanied by an increase in the memory T cells’ pool, poised for its efficient role upon reinfection. Nevertheless, some patients undergo immunosuppression and are susceptible to secondary infections due to a cellular immune response defect. Prolonged exposure to parasite antigens and chronic inflammation influence different functions of memory T cells, associated with immunopathology in VL. This phenomenon, identified as T-cell exhaustion, has variable effects on the disease outcome depending on the species of *Leishmania* involved (117). Recently, exhausted T-cell subsets have also been characterized in experimental CL caused by *L. mexicana* based on CXCR5 and TIM-3 (T-cell immunoglobulin and mucin domain-containing protein 3) expressions (118).

## 4 Memory T-cell subtypes and protection against the *Leishmania* infection

Effector T cells, once they migrate to the tissues, tend to have a short lifespan, but memory T cells are defined by their long-term presence following the resolution of an infection (119). The hallmark of T-cell memory is the ability to mount a robust and rapid response upon secondary challenges with a previously recognized pathogen (120–122). Memory T lymphocytes encompass heterogeneous populations, identified through surface markers and their specific functions, such as production of cytokines and their ability to proliferate (123–126) (**Table 2**). In general, three primary subsets of memory T cells have been identified, comprising circulating effector memory T cells (T_EM_) found in non-lymphoid tissues, circulating central memory T cells (T_CM_) located in secondary lymphoid organs, and non-circulating tissue-resident memory T cells (T_RM_) capable of persisting in non-lymphoid tissues.

**Table 2:** Markers commonly used to differentiate memory T cells.

Markers	Type/Function	Memory T cells			Naïve T cells	
*β1 integrin (CD29, CD49d, CD 49e)*	Adhesion molecules	High			Low	
*β2 integrin (CD11a, CD11b. CD18)*	Adhesion molecules	High			Low	
*CD2*	Adhesion/activation molecules	High			Low	
*CD44*	Adhesion molecules	High			Low	
*CD54*	Adhesion molecules	High			Low	
*CD58*	Adhesion/activation molecules	High			Low	
*Ly-6C*	Intercellular adhesion	High			Low	
*IL-2R β-chain (CD122)*	Receptor components	High			Low	
		Naïve T	T_EFF_	Memory T cells		
				T_EM_	T_CM_	T_RM_
*CD45RA*	Receptor/signaling	+	+	–	−	–
*CD45RO*	Receptor/signaling	−	–	+	+	+
*CD62L*	Lymph node homing via HEVs	+	Low	Low	+	
*CCR7*	Lymph node homing via HEVs	High	Low	High	High	
*IL-7Ra (CD127)*	Receptor	+	−	Low	High	
*CD95 (Fas)*	Receptor/cell death	−	High	High	High	
*CD27*	Costimulatory marker	High	−	Low	+	
*CD28*	Costimulatory marker	+	Low	Low	High	Low
*CD57*	Differentiation marker	−	+	Low	−	
*PD-1*	Negative costimulatory receptor	−	Low	+	−	Low
*CD69*	Activation marker	−	+	Low	−	+
*CD103 (αE integrin)*	Epithelial homing	−		+		+

T_EM_, effector memory; T_EFF_, effector; T_CM_, central memory; T_RM_, resident memory; HEVs, HIGH endothelial venules. + shows presence and - shows absence or very low expression.

Studies suggest that following an acute viral infection, memory CD8^+^ T cells do not need antigen for their survival, keep the capacity for homeostatic proliferation, and exhibit robust responsiveness to IL-7 and IL-15 cytokines. However, in the chronic infection, CD8^+^ T cells induced by viral infection are unable to acquire the feature of memory T cells to persist over the long term without requiring antigenic stimulation (127). Nevertheless, the development and maintenance of memory CD4^+^ T cells might be obviously different (128) and the nature of CD4^+^ memory responses is likely to vary depending on variations in antigenic exposure and persistence (129).

### 4.1 T_EM_/T_CM_ subpopulations

The study of Lanzavecchia and colleagues demonstrated that memory T cells in human could be categorized into two subpopulations based on the expression of molecules governing T-cell migration (73). Some memory T cells express molecules required for migration to lymph nodes including CCR7 and CD62L, and are named T_CM_. CD62L, also known as L-selectin, is a C-type calcium-dependent lectin of the selectin subfamily expressed on most leukocytes. The most important function of CD62L is participation in lymphocyte homing to secondary lymphatic organs (130). Other memory T cells lack markers for lymph node homing and are able to migrate to various tissues. Notably, T_CM_ cells do not produce effector cytokines like IFN-γ, whereas in peripheral tissues, T_EM_ are capable of producing effector cytokines. Similarly, studies in mice have suggested the existence of heterogeneous populations of memory T cells with diverse migratory potentials and effector functions (119, 131).

There are several other surface, costimulation, and activation markers that might be used to distinguish the memory T-cell subpopulations (132) (**Table 2**); among them are CD27, belonging to the tumor necrosis factor/nerve growth factor receptor (TNF/NGF-R) family, and CD28, a member of the Ig superfamily required for both CD4^+^ and CD8^+^ memory T-cell expansion (133, 134).

#### 4.1.1 T_EM_/T_CM_ cells in leishmaniasis: mouse studies

Mice that have successfully recovered from an initial *Leishmania* infection display robust immunity to reinfection. To investigate the T cells responsible for this protection, CD4^+^ T cells from mice that recovered from *L. major* infection were purified and transferred to naive mice. Analysis revealed two distinct subpopulations of protective T cells in immune mice (135). One subset exhibited the characteristics similar to effector T cells, such as low expression of CD62L, capacity to induce DTH, production of IFN-γ, and lack of migration to the adjacent lymph nodes.

Another subset that exhibited characteristics of central memory T cells were CD62L^hi^, with high IL-2 but no IFN-γ production. They migrated to the draining lymph node and proliferated upon challenge, which is notably accompanied by downregulation of CD62L expression, acquiring IFN-γ production capacity, and migration to infection sites (70). Adoptive transfer of sorted CD62L^hi^ and CD62L^low^ CD4^+^ T-cell subpopulations to naïve mice showed that both populations of T cells conferred protection upon a subsequent infection. The ability of T_CM_ cells (CD62L^hi^) to confer protection is aligned with the observation that upon a subsequent *L. major* challenge the lymph node-homing T_CM_ cells can expand and become T_EFF_ cells that proliferate and produce IFN-γ at the site of infection (70, 135).

Experiments with engineered thymidine auxotrophic *L. major* strain (*dhfr-ts*), which fails to survive long term *in vivo* (136), showed that following the eradication of the parasites, a group of *Leishmania-*specific CD4 T cells emerges, characterized by the T_CM_ phenotype (CD62L^hi^, CCR7^hi^) (136, 137). Remarkably, these cells can persist even when *Leishmania* parasites are no longer present. Consequently, existence of at least two distinct circulating subpopulations of *Leishmania*-specific CD4^+^ T cells are suggested in *L. major*-infected mice. One subset, referred to as T_EFF_ cells, lacks the ability to survive without persistent parasites, while another population named T_CM_ cells that emerges early post-infection displays the ability to survive once the parasites are eliminated. Some evidence suggests that the CD4^+^ T_CM_ pool is heterogeneous with the potential to develop into either one of Th1 or Th2 cells (138, 139). Immunization of mice with a centrin gene-deleted strain of *L. donovani* (*LdCen−/−*) induced increased CD4^+^CD62L^hi^, CCR7^+^ T_CM_ cell (71) and CD8^+^CD62L^+^CD27^+^ T_CM_ cell responses (72), which, upon restimulation with antigen *in vitro*, differentiated into T_EFF_ cells.

#### 4.1.2 T_EM_/T_CM_ cells in leishmaniasis: human studies

Despite the fact that substantial knowledge exists regarding the development of primary effector T-cell responses and memory T-cell development in the murine model of *Leishmania* infection, the generation and maintenance of memory T cells in human leishmaniasis are less understood [14, 15]. Individuals with a history of cutaneous leishmaniasis (HCL) who have acquired protection against further infections provide a suitable population for investigating the potential role of memory T cells in protection against leishmaniasis. To this end, in tandem with experiments conducted in mouse model, we focused on HCL volunteers to study distinct subsets of CD4^+^ and CD8^+^ memory T-cell subpopulations and to understand the protective role of memory T cells in human CL. We have characterized different T cells from the blood of HCL and control volunteers and have shown that stimulation of sorted cells with *Leishmania* antigen in culture significantly increased CD4^+^CD45RA^−^CCR7^−^ T_EM_ cells from CD4^+^CD45RA^+^CCR7^+^ naïve T cells, and by carboxyfluorescein diacetate, succinimidyl ester (CFSE) labeling, CD4^+^CD45RA^−^CCR7^+^ T_CM_ cells exhibited a greater capacity to proliferate compared to CD4^+^ T_EM_ cells. Typically, both CD4^+^ and CD8^+^ T cells quickly engage in a proliferative response following a brief exposure to antigen stimulation. The phenotypic change of naïve cells to memory cells and commitment of T_CM_/T_EM_ cells to high proliferation may guarantee the formation of a substantial memory T-cell pool, even when antigen levels decline in later stages of the immune response (140).

For the first time in human leishmaniasis, in individuals with a history of CL, a mixture of *Leishmania*-responsive CD4^+^ T_EM_ cells capable of producing IFN-γ and *Leishmania*-responsive CD4^+^ T_CM_ cells capable of producing IL-2 was detected. These cells may contribute to protective immune response against *Leishmania* infection (74). In a parallel study, the T-cell subset composition was assessed in peripheral blood CD8^+^ T cells obtained from individuals with HCL. When sorted CD8^+^ memory T cells from HCL volunteers were stimulated *in vitro*, there was a notably elevated levels of IFN-γ production in comparison to cells of healthy controls. A similar result was consistently observed in intracellular IFN-γ staining as well. The memory population was found to be accountable for the IFN-γ production triggered by *Leishmania* in culture. Among the subsets, *Leishmania*-responsive proliferating CD8^+^ T_EM_ cells were the most prevalent, and they could potentially contribute to protection in the company of CD4^+^ T cells (44). Recently, the frequency of T_CM_ and T_EM_ cells during active CL and post-treatment has been analyzed in patients with *L. braziliensis* infection (75), and a higher frequency of CD4^+^ T_EM_, CD8^+^ T_EM_, and CD8^+^ T_CM_ during CL development and following treatment has been observed (75).

The frequency and function of central and effector memory CD4^+^ and CD8^+^ T cells have also been investigated in New World CL patients due to *L. braziliensis*. In two separate studies, both CD4^+^ and CD8^+^ T_EM_ cells were found to be higher than T_CM_ or naive T cells in the skin lesions (76) and blood mononuclear cells following antigenic stimulation in active CL patients (34). Another recent study in Brazil assigns an even greater role to the CD8 compartment, showing that there is an increase in CD4^+^ T_EM_ during CL, and CD8^+^ T_EM_ and CD8^+^ T_CM_ during CL and post-treatment (75), while it was previously suggested that CD4^+^ and CD8^+^ T_EM_ compartments from healed CL individuals decreased gradually with time after clinical cure (141).

In an *ex vivo* study, a lower frequency of blood CD4^+^CD45RO^+^ memory cells without stimulation, and fewer CD4^+^ CD25^hi^FOXP3^+^ T_REG_ cells after antigenic stimulation of mononuclear cells from symptomatic VL patients compared to recovered VL subjects were demonstrated, showing the immune impairment during symptomatic VL caused by *L. infantum* (77). However, the frequencies of the CD4^+^ and CD8^+^ T_CM_ cells, responsible for producing IL-2, TNF-α, and IFN-γ, were notably elevated in the blood of VL patients following treatment (78).

### 4.2 T_RM_ subpopulation

**Figure 1:**
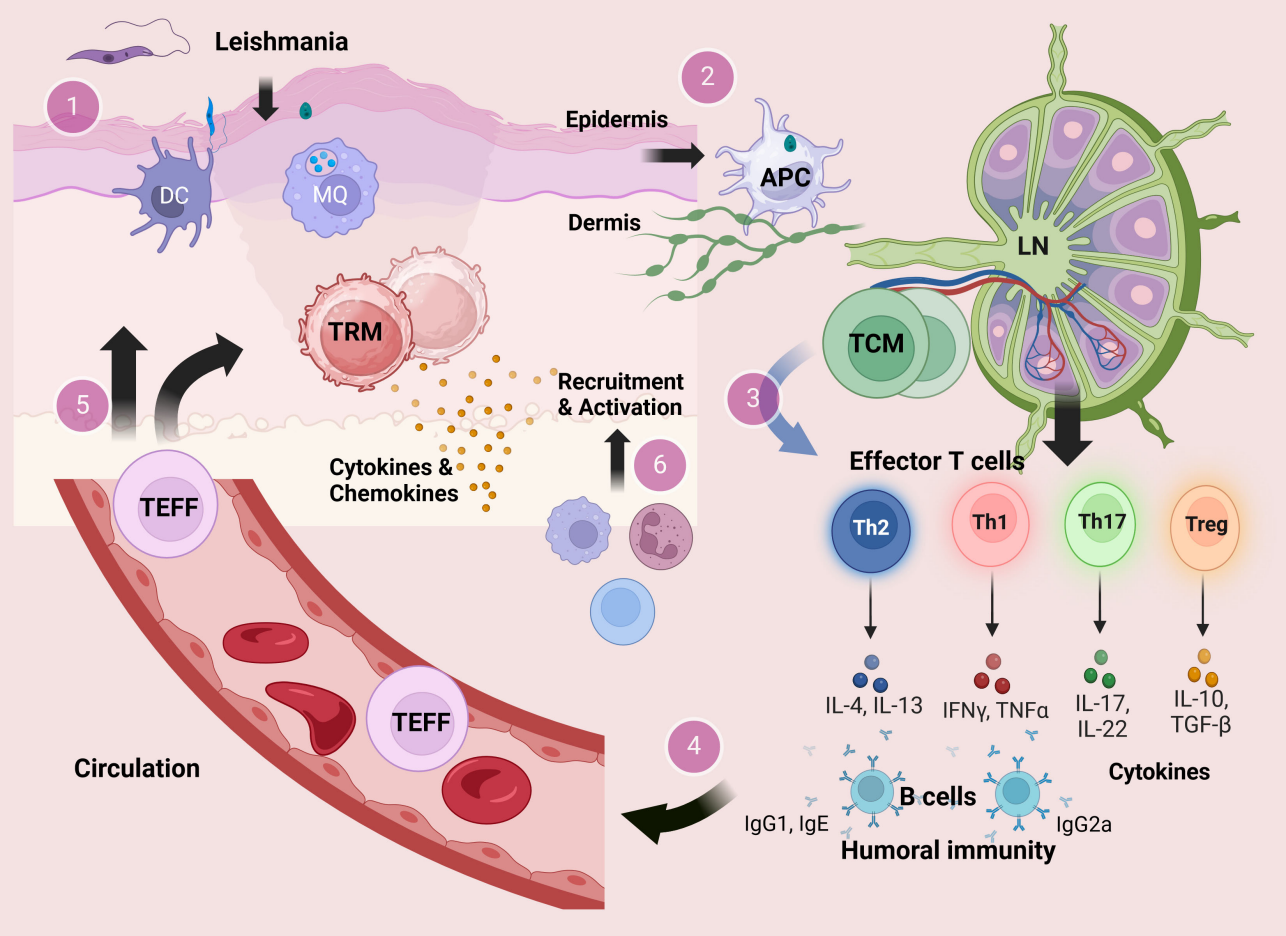
The development of T cells and their contribution in the immunity during experimental cutaneous *Leishmania* infection. At the site of sand fly bite, *Leishmania* metacyclic promastigotes infect cells of the monocyte–macrophage lineage including macrophages and DCs in the skin dermal layer. Within the host cells, promastigotes transform to the amastigote stage and replicate by binary fission (1). Infected antigen-presenting cells (APCs) carry antigens to lymphoid organs leading to activation of CD4^+^ T cells in the draining lymph nodes (2). Activated CD4^+^ T cells in the draining lymph nodes could develop into either effector T (T_EFF_) cells or central memory T (T_CM_) cells. In the course of secondary infection, the residing T_CM_ cells could be reactivated and differentiated into different subsets of CD4^+^ T_EFF_ cells (3) that provide a circulating pool of Leishmania-reactive T cells (4). Circulating CD4^+^ T_EFF_ cells can be rapidly recruited into the site of infection, while some T_EFF_ cells may migrate into skin distant from the lesion site and become resident memory T (T_RM_) cells (5). In response to challenge, T_RM_ cells in the skin produce cytokines that mediate recruitment of inflammatory monocytes and effector T cells from the blood (6).

In **Figure 1**, the development of different T-cell subsets and their contribution in the immunity during experimental cutaneous *Leishmania* infection in mice are outlined (**Figure 1**). Following activation, a considerable fraction of T cells remains in circulation, while a distinct group of these activated T cells migrates into tissues, taking on a resident role as T_RM_ cells. Among CD8^+^ T-cell compartments, the T_RM_ cells are described as a subset that can be found in nearly all tissues of the body and are responsible for either defending against infections or occasionally triggering autoimmune responses (142, 143). There is growing support for the significant contribution of T_RM_ cells in the relapse of chronic inflammatory diseases of the skin (144). On the other hand, compared to CD8^+^ T cells, knowledge regarding CD4^+^ T_RM_ cells is limited, which have been identified in various tissues such as lungs, intestines, and skin (145).

**Figure 2:**
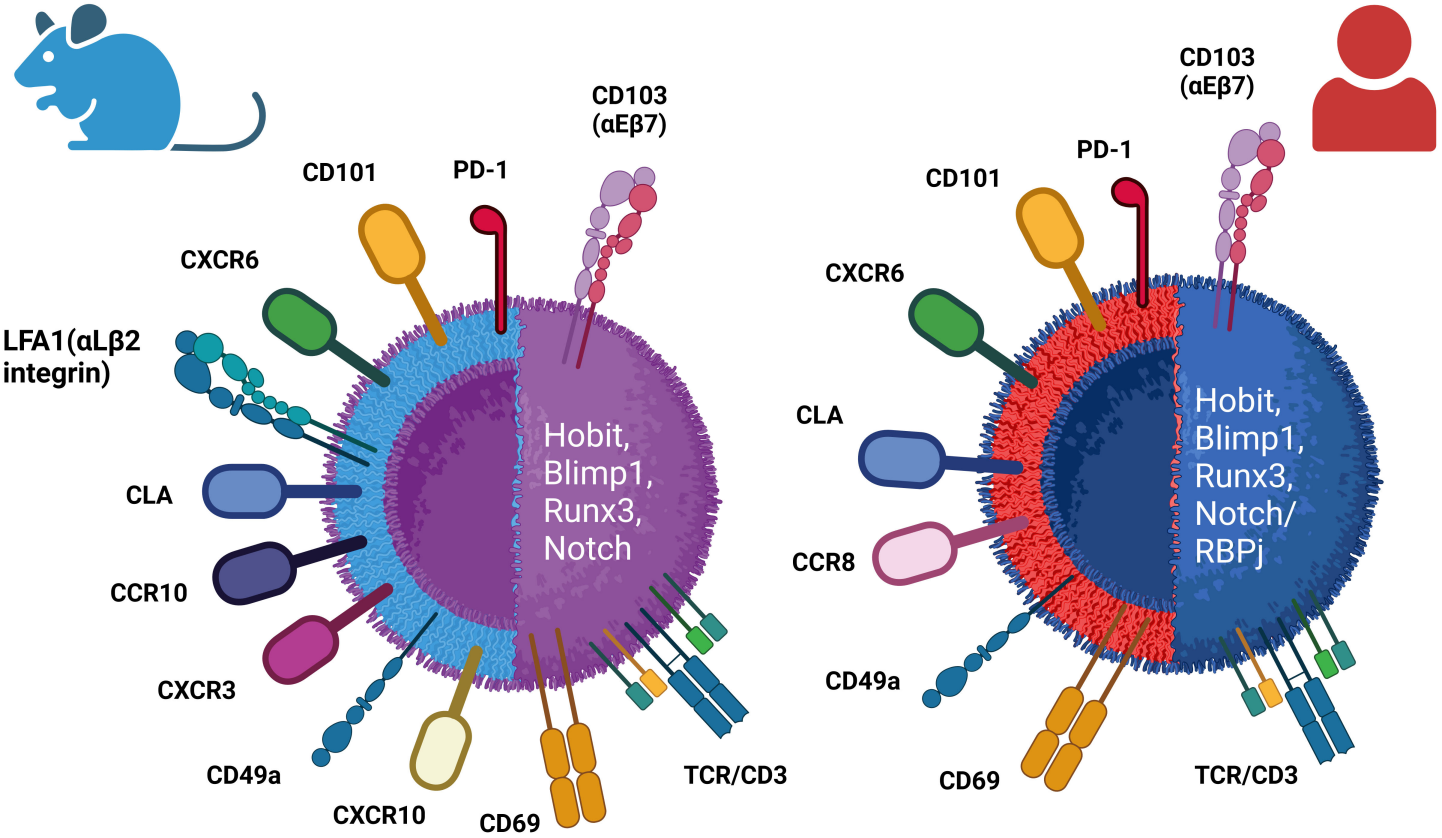
Differential phenotypes of mouse and human resident memory (T_RM_) cells. T_RM_ cells are identified by the expression of canonical receptors CD103 and CD69, as well as several other surface markers depending on the specific resident tissues. T_RM_ cells display upregulation of the expression of some transcriptional factors, including *Hobit, Blimp1, Runx3*, and *Notch* (/*RBPj*).

T_RM_ cells are primarily characterized by high expression of canonical markers’ CD69 adhesion molecule and the αE integrin CD103 and downregulation of homing receptors CCR7 and CD62L. Various other markers, such as CXCR3 and CD49a, are also used to identify T_RM_ cells in specific tissues (146). In **Figure 2**, a comparison between mouse and human tissue-resident memory T cells regarding the expression of surface markers and transcription molecules is illustrated (**Figure 2**) (147). The increased expression of four transcription factors plays a crucial role in the development and maintenance of T_RM_ cells, including *Runx3, Notch, Hobit*, and *Blimp1* (148–150).

It was suggested that following an acute viral infection, CD8^+^ T cells are promptly recruited to the skin, a process facilitated by CD8^+^ T cells expressing E- and P-selectin ligands (151). Upon reaching the skin, additional factors facilitate the retention of T cells in the tissue. CD69 is a type II C-lectin receptor found on cell membranes of hematopoietic stem cells, T cells, and several other cells of the immune system. It is also implicated in T-cell differentiation as well as lymphocyte retention in lymphoid organs. CD69, as an early marker of T-cell activation, is detected in a substantial fraction of tissue-resident memory CD4^+^ and CD8^+^ T cells across both murine and human. The continuous expression of CD69 on T_RM_ cells restricts tissue exit by antagonizing sphingosine-1-phosphate receptor 1 (S1PR1)-mediated extravasation. The presence of S1PR1, alongside the transcription factor Kruppel-like factor 2 (*KFL-2*), stimulates T-cell migration and is rather characteristic of circulating cells (152–154). It was demonstrated that in human, the transcriptional profile of the CD69^+^ subset of memory CD4^+^ and CD8^+^ T cells is distinct from that of CD69^−^ memory cells in tissues and circulation (147, 149).

On the other hand, CD103, a heterodimeric transmembrane surface receptor, is an integrin that binds E-cadherin on epithelial cells, exhibited by some CD8^+^ memory T cells in mucosal tissues (155, 156). In both humans and mice, CD8^+^ T_RM_ cells located in mucosal tissues including the skin, lungs, salivary glands, and small intestines are characterized by a notable enrichment of CD103 expression (157–160). The function of CD103 on CD4^+^ T_RM_ cells is not fully understood, since a significant portion of CD4^+^ T_RM_ cells in both mouse and human usually do not express the CD103 marker (161, 162), albeit, in specific tissue locations such as the skin in mice, populations of CD103^+^CD4^+^ T_RM_ cells have been identified (163).

In mice, the skin harbors CD103^+/−^CD4^+^ T_RM_ as the most frequent population (163), although significant quantities of CD103^+^CD8^+^ T_RM_ could be found in epidermis post-infection (156). In healthy human skin, the majority of T_RM_ cells located in the dermis express CD69, but not CD103 (CD4^+^CD69^+^CD103^–^ cells), while in the epidermis layer, mixed populations of CD4^+^ and CD8^+^ T_RM_ expressing CD103 are found (159), which show robust effector capabilities coupled with restricted proliferative potential in contrast to CD103^−^ T_RM_ cells. Elevated levels of cutaneous lymphocyte antigen (CLA) and distinct chemokine receptors like CCR4 have been observed in these T_RM_ cells. Additionally, human skin contains T_RM_ cells that express the chemokine receptor CCR8 upon infiltration into the skin (164, 165), and these CCR8-positive T cells exhibit all the predefined characteristics of resident memory T cells (**Table 2**; **Figure 2**).

Little is known about the transcriptional regulation and signaling pathways involved in the long-term maintenance of T_RM_ cells in different tissues. IL-7 and IL-15 have an established role in controlling homeostatic proliferation and survival of memory T cells (166, 167). Increased levels of IL-7 and IL-15 receptors were demonstrated on T_RM_ cells, similar to circulating memory T cells, implying that these cytokines might also play a role in survival of T_RM_ cells (168). In the skin, the local mediators such as transforming growth factor (TGF)-β, IL-7, and IL-15 are required for regulation of long-lived CD8^+^ T_RM_, whereas IL-7 contributes to the persistence of CD4^+^ T_RM_ (169). It seems that T_RM_ in different tissues may rely on various signals to support their persistence and survival (147).

#### 4.2.1 T_RM_ cells in leishmaniasis: mouse studies

In experimental leishmaniasis, lesions harbor both CD4^+^ T cells that lack CD69 expression presumably in traveling through the tissues and a separate group of resident T cells expressing the CD69 marker (113). An experiment with *L. major* infection in the mouse model showed that residency-associated transcription factors *Blimp-1* and *Hobit* (148) were more predominantly found in CD69^+^ T cells within leishmanial lesions as opposed to CD44^+^ T cells in the draining lymph nodes, whereas sorted CD4^+^CD69^+^ T cells from the lesion expressed a low level of both *S1pr1* and *Klf2* factors (113). This result suggested that CD4^+^CD69^+^ T cells are preferentially located in cutaneous *Leishmania* lesions as skin T_RM_ cells. In leishmaniasis, the skin houses resident CD4^+^ T cells capable of producing IFN-γ upon restimulation (64). Following a primary infection with *L. major*, *Leishmania*-reactive Th1 effector T cells undergo proliferation within the draining lymph nodes and then migrate to the infection site, but they are not restricted to the infection site and can migrate to non-inflamed regions throughout the skin. A compartment of these cells transforms into CD4^+^ T_RM_ cells, which in the secondary challenge infection produce IFN-γ to activate macrophages. The activated T_RM_ cells also produce chemoattractant mediators like CCL2 and CCL7 [chemokine (C-C motif) ligand 7], which induce recruitment of inflammatory monocytes (65, 170). In secondary *Leishmania* infection, upon IFN-γ stimulation, this monocyte population promptly initiated the production of inducible nitric oxide synthase (iNOS), which activates parasite killing mechanisms (65, 171). Therefore, skin-resident memory CD4^+^ T cells protect against *L. major* challenge in mice and they are independent of persistent parasites for survival (65, 113).

In VL infection in animal models, resolution of *Leishmania* infection is dependent on the development of T cell-mediated immunity associated with the formation of granulomas in the liver. Development of immune memory subsets in the liver-resident T-cell population in response to *L. infantum* antigens has been suggested in experimental infection in BALB/c mice and treatment with meglumine antimoniate has shown to improve hepatic immune response by increasing the levels of effector and memory T cells (68).

#### 4.2.2 T_RM_ cells in leishmaniasis: human studies

Although the CD69 marker has been assessed as an activation marker in CD4^+^ and CD8^+^ T-cell compartments in blood and skin lesion of human leishmaniasis patients (76, 141), insufficient data are available regarding the involvement of T_RM_ cells in human leishmaniasis.

Since the resolution of CL lesion and VL disease typically coincides with the development of a protective immune response and lifelong resistance to reinfection in human (30, 43, 172), further insight into the characterization of memory T-cell phenotypes and functions holds promise for using memory cells as potential biomarkers of protection and as a tool for assessing the effectiveness of candidate vaccines against leishmaniasis.

## 5 Biomarkers of immune protection in leishmaniasis

A key obstacle in the development of a leishmaniasis vaccine lies in the identification of reliable surrogate marker(s) for evaluating immune protection in human (6). The biomarkers might have different applications including evaluation of innovative diagnostic tools and monitoring the disease progression or the outcome of clinical interventions (173). In order to evaluate vaccine efficacy as a preventive intervention against human leishmaniasis, the researchers are required to clearly define measurable biomarkers as indicators of protection (4).

CD4^+^ Th1/Th2 population subtypes and their secreted cytokines in *Leishmania* infection has long been investigated and introduced as a putative biomarker of immunity against *Leishmania* infection (174). Several decades ago, Kellina observed differences in the susceptibility of various strains of inbred mice to *L. major* infection (175) and Mitchell showed that in the course of *L. major* infection in BALB/c nu/nu mice, the outcome may be either a progressive or a resolving lesion, contingent upon the quantity of T cells utilized for reconstitution (176). The role of CD4^+^ T cells in either resistance or susceptibility of different strains of mouse to infection with *L. major* has been established by several groups in the early 1980s (177). Mossman and Coffman defined two functionally distinct CD4^+^ T-cell subsets, Th1 and Th2, based on their functions and cytokine secretions (178, 179), and others explained differential function of Th1 and Th2 CD4^+^ subsets in the outcome of murine *L. major* infection: mice that effectively overcome the infection primarily demonstrate a Th1 type of immune response with high levels of IFN-γ production but little IL4, and mice with fatal, disseminating disease have a Th2 type of immune response, including a significant amount of IL4 and IL5 and lower levels of IFN-γ (177, 180–182). Although Th1/Th2 dichotomy is well established in the *L. major* infection model in the resistant C57BL/6 and susceptible BALB/c strains of inbred mice, this paradigm seems to be more complex in humans (183) and might be different in mice with other genetic backgrounds and in using other parasite strains (184–187). In addition, existence of non-cure phenotypes in spite of strong Th1 response suggested additional involving mechanisms (23, 188). Moreover, although some vaccines induce Th1-type cytokines, they have no or only a small effect on organ pathology (188) and protection could be achieved without activating a strong Th1 response (139).

The immune response to *Leishmania* infection in human is more complicated than what is usually retrieved from experiments in inbred animal models, and studies have yet to definitively demonstrate a clear-cut Th1/Th2 phenotype in CL (189). There are studies suggesting an association between Th1/Th2 dichotomy and the outcome of human leishmaniasis. It has been shown that in symptomatic VL and active DCL patients, a Th2 response was induced (190, 191), whereas a strong expression of IFN-γ and low IL-10 levels was observed in healing CL patients and after healing of skin lesions following treatment in DCL patients (30, 43, 172, 190, 192). Evidence shows that both antigen-specific IFN-γ-producing CD4^+^ and CD8^+^ T cells contribute to the immunity against human leishmaniasis, though the role of CD4^+^ T cells with two arms of Th1 and Th2 cells has more been highlighted in the outcome of disease (43, 193).

IL-10 produced by T_REG_ cells has been identified as a factor that dampens both the intensity and effectiveness of the Th1 response (194), but despite this suppressive effect that leads to disease progression and parasite persistence, IL-10 is an indispensable immunoregulator that inhibits an exacerbated immunopathology and tissue damage caused by increased production of inflammatory cytokines, especially IFN-γ in CL (195).

It is believed that in human VL, while the majority of the infected individuals often experience a subclinical or asymptomatic infection accompanying robust cellular immune response, healing or protective responses are associated with the production of Th1-type cytokines, including IFN-γ, and conversion in the leishmanin skin test (LST) (196, 197). However, there are reports suggesting that VL might be exacerbated even when measurable amounts of Th1 cytokines are present, which are functionally neutralized by the function of other mediators, such as IL-10 (198, 199). Active VL is characterized by suppressed cell-mediated immunity, shown by a negative LST response, which is typically converted to positive in patients after cure or recovery from VL (200).

## 6 Memory T cells as putative biomarkers for protection and efficacy assessment of candidate vaccines

As mentioned above, the challenge in developing effective leishmaniasis vaccines lies in the identification of surrogate markers for protection. Active effector T cells, although vital in immune responses, are short-lived and require continuous stimulation, making them less favorable for vaccination strategies. However, recent advancements in understanding memory T cells, particularly parasite-independent subsets, raised new hope in this way.

Among these memory T-cell subsets, T_CM_ and skin T_RM_ have emerged as key players in *Leishmania* infection in mouse models. T_RM_ cells, a population of CD4^+^ T cells resident in the skin, play a significant role in concomitant immunity against cutaneous *Leishmania* infection. They respond to challenge infection by rapid recruitment at infection site and producing IFN-γ upon restimulation, effectively reducing the parasite burden. A crucial aspect of *Leishmania*-reactive T_RM_ cells is their potential to exist not only at the infection site but also in noninflamed skin regions distant from the lesions. This feature enables them to provide protection against challenge infections throughout the skin, even in the absence of *Leishmania*-specific T_EFF_ cells. Therefore, since CD4^+^ T_RM_ cells are long-lasting memory subsets independent of persistent parasites, successful vaccine strategies may include induction of CD4^+^ T_RM_ cells as efficient immunogenicity outcome. In addition, assessing the induction of CD4^+^ T_RM_ cells could be used as a biomarker of immune protection against leishmaniasis in evaluating the efficacy of candidate vaccines. While there are examples of successful leishmaniasis vaccines that have demonstrated strong immunogenicity in both humans and animal models, so far, there is no vaccine available for any type of human leishmaniasis (6). This challenge is not exclusive to leishmaniasis but extends to many infections reliant on sustaining a memory T-cell response (113). Recently, development of a series of live attenuated centrin gene-deleted *Leishmania* strains, including *L. donovani*, *L. mexicana*, *L. braziliensis*, and *L. major*, by using the CRISPR/Cas9 method, has shown promise in inducing protection against challenge infection (201). Mice immunized with a marker-less *Lm*Cen^−/−^ *L. major* developed no evident lesions following challenge with the bite of *L. major*-infected sand flies (66). This protection is attributed to IFN-γ-secreting CD4^+^ T_EFF_ cells, and immunization using *Lm*Cen^−/−^ further triggered the development of CD4^+^ T_RM_ cells in the skin, accompanied by the production of cytokines and chemokines required for their prolonged persistence, resembling the effects of leishmanization (67). Upon challenge with wild-type *L. major*, specific T_RM_ cells were rapidly recruited and underwent proliferation at the infection site and triggered substantial production of IFN-γ and granzyme B in immunized mice compared to control mice (67). Similarly, immunization with the *Lmex*Cen^−/−^ *L. mexicana* strain confers protection against challenge infection of wild-type *L. mexicana* parasites in both BALB/c and C57BL/6 mouse models (202). Interestingly, the same mutant strain (*Lmex*Cen^−/−^) also induced long-term protection against *L. donovani* VL infection in a hamster model (203). Mice immunized with *LmexCen−/−* exhibited a markedly elevated proportion of CD4^+^CD44^+^CL62L^+^ T_CM_ cells in their lymph nodes compared to control mice injected with PBS, and this population of CD4^+^ memory T cells might contribute to long-term protection against *L. mexicana* infection (202).

## 7 Discussion

Over the last few years, a growing body of evidence has emerged, suggesting that T_RM_ cells have both protective and pathogenic roles in different infectious diseases and immunological disorders (204). Our current knowledge implies that T_RM_ cells serve as a first line of defense in peripheral tissues to protect human against different pathogens, including viruses, intracellular bacteria, and protozoan parasites [reviewed in (205)].

In *Plasmodium* infection, CD8^+^ T_RM_ cells appear to play pivotal roles in protective immunity to liver-stage malaria, which is critical for vaccine development because parasites must be eliminated at the liver before development of blood-stage infection to prevent clinical manifestations of malaria (206). Induction of liver-resident memory T cells associated with protection has been shown in several experimental vaccine platforms including radiation-attenuated sporozoite (RAS) and the circumsporozoite protein (CSP)-based vaccines and a recent mRNA-containing lipid nanoparticle (mRNA-LNP) formulation (207–209). The protective role of T_RM_ cells has also been investigated in *Toxoplasma gondii*, the intracellular protozoan parasite causing toxoplasmosis, which forms persistent cysts in various tissues in human and mammals. In a chronic infection model of *T. gondii* infection, the CD103^+^ CD8^+^ T_RM_ phenotype accumulated within the brain and produced both IFN-γ and TNF-α cytokines, which are critical for parasite control within the central nervous system (CNS). It was suggested that a higher protective immunity was conferred by brain T_RM_ against *T. gondii* infection in the CNS when contrasted with the protective capabilities of CD8^+^ T_EM_ and T_CM_ cells (210). Similarly, an experimental study on a mouse model of *Trypanosoma (b.) brucei* infection suggests that in the early stages of disease, most brain T cells exhibit a T_EM_ phenotype, while during the late cerebral stage, T cells show a T_RM_ phenotype and may have a potential role in causing sleep disorder during trypanosomiasis (211).

These accumulating lines of evidence on the contributing role of T_RM_ cells in the pathogenesis of protozoan parasites suggest that exploring methods to generate and sustain T_RM_ cells is essential for the advancement of vaccine development against parasitic infections.

In mice *Leishmania* infection, following resolution of leishmanial lesion, there are circulating T_EFF_ cells that are capable of offering protection upon challenge, yet they fail to survive when parasites are no longer present. While vaccine formulation based on the permanent delivery of parasitic antigens to the immune system for induction of circulating T_EFF_ cells that could provide protection to challenge is not a feasible strategy, vaccines could target antigen-independent memory CD4^+^ T cells subsets including long-lived skin-resident memory T cells that have the capacity for long-term maintenance in the absence of persistent parasites. Further research is required to characterize the specific T-cell populations and pathways activated by this type of vaccination, but in experiments with *L. major* infection in the murine model, the immunization led to the expansion of CD4^+^ T cells in the draining lymph node, which subsequently migrated to unaffected skin areas through circulation and differentiated into T_RM_ cells. Experimental immunization has been successful in the induction of T_RM_ cells that confer long-term protection against reinfection (67, 212). While a human vaccine for leishmaniasis remains unavailable, successful examples of experimental vaccines suggest that inducing protective, long-lived skin CD4^+^ T_RM_ cells, independent of persistent parasites, might be a promising approach for future vaccine development and evaluating the protective efficacy induced by such vaccination (213).

**Figure 3:**
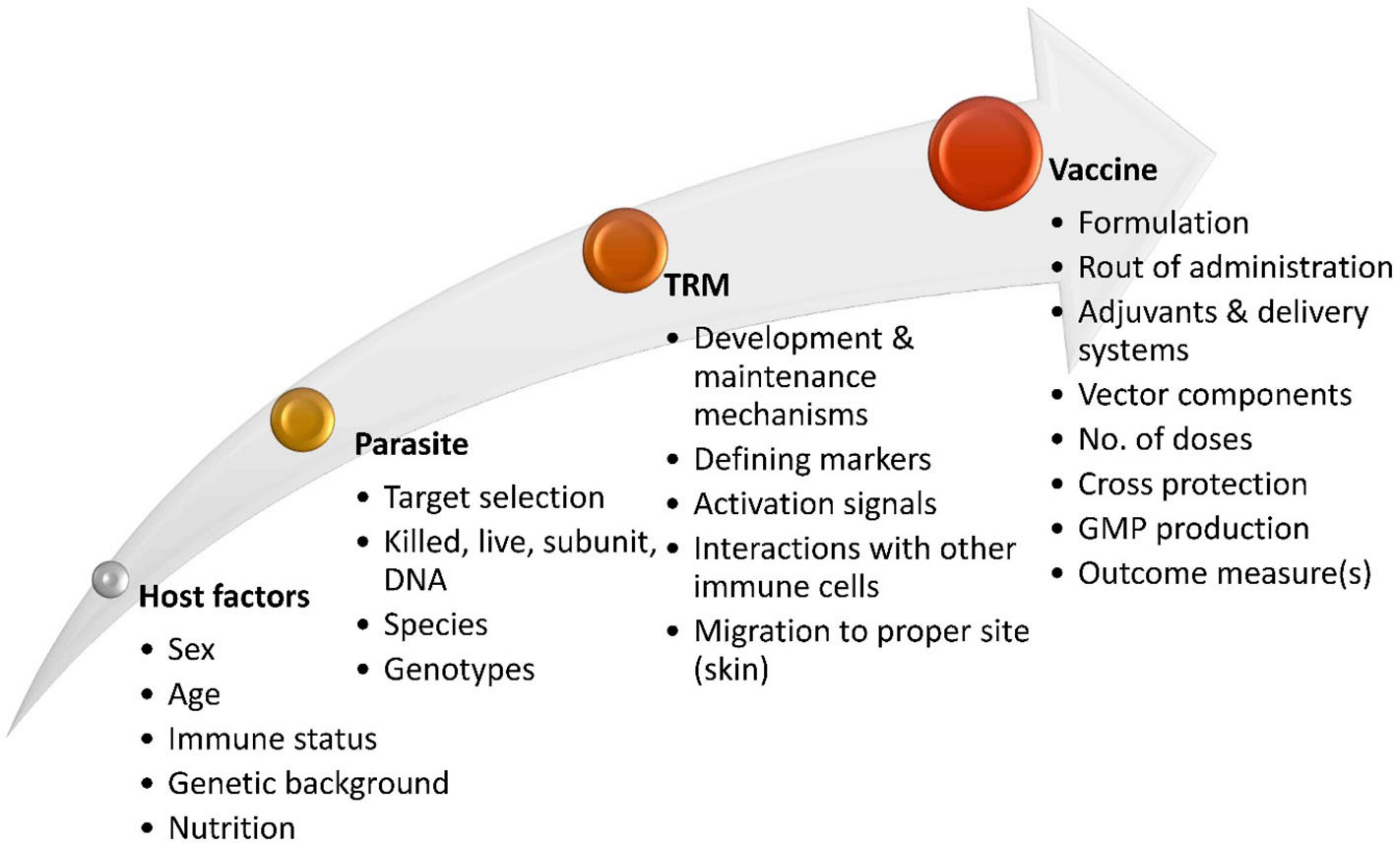
Factors involved in the development of *Leishmaniasis* vaccines aimed at inducing skin-resident memory T cell response. Effective vaccine strategies for inducing T_RM_ cell responses in leishmaniasis will rely on a comprehensive understanding of the development and maintenance of protective T_RM_ cells in *Leishmania* infection. Exploring the potential migration and localization of vaccine-induced T_RM_ cells in specific sites in the skin and the signals needed to activate efficient T_RM_ cells in an interactive microenvironment with other immune cells is also imperative. Various other factors of *Leishmania* parasite, host background, and vaccine design are involved in the effectiveness of candidate vaccines targeting the generation of protective T_RM_ cells.

The approval of a vaccine for human leishmaniasis still encounters significant challenges (214) and a more comprehensive understanding of the development and maintenance of protective memory T cells is necessary. Various parameters need to be taken into consideration to optimize vaccine formulations in leishmaniasis targeting skin tissue-resident memory responses (**Figure 3**). Defining outcome measures and finding biomarkers that correlate with T_RM_ responses could provide a straightforward approach to assess skin-resident memory responses following vaccination in humans, as previously suggested (215).

Given the encouraging and important result obtained concerning the role of T_RM_ cells in the protection of immunized mice against *Leishmania* infection, they might be used as potential biomarkers of protection in the assessment of the efficacy of candidate vaccines in human leishmaniasis. However, before we could apply memory T cells as a biomarker, numerous critical questions remain to be answered, including the development of tissue-resident memory T-cell subsets, the possible differences between CD4^+^ and CD8^+^ memory T-cell subsets, and finally the role of persistent parasite in T_RM_ survival in human leishmaniasis.

## Author contributions

MN-R: Writing – original draft, Writing – review & editing, Conceptualization. YS: Writing – review & editing.
